# Supplementary material for: High Tumor-Infiltrating Lymphocyte Count Is Associated with Distinct Gene Expression Profile and Longer Patient Survival in Advanced Ovarian Cancer
Source: Int J Mol Sci. 2023 Sep 5;24(18):13684. doi: 10.3390/ijms241813684 (PMC10530512; doi:10.3390/ijms241813684)
Supplement: Supplementary file 1 [file ijms-24-13684-s001.zip › ijms-2562375_Suppl_Figs,_Tables_1_2_and_Methods.pdf]

Andras Jozsef Barna, Zoltan Herold, Miklos Acs, Sandor Bazsa, Jozsef Gajdacsi, Tamas Marton Garay, Magdolna Herold, Lilla Madaras, Dorottya Muhl, Akos Nagy, Attila Marcell Szasz† and Magdolna Dank†

High Tumor-Infiltrating Lymphocyte Count Is Associated with Distinct Gene Expression Profile and Longer Patient Survival in Advanced Ovarian Cancer

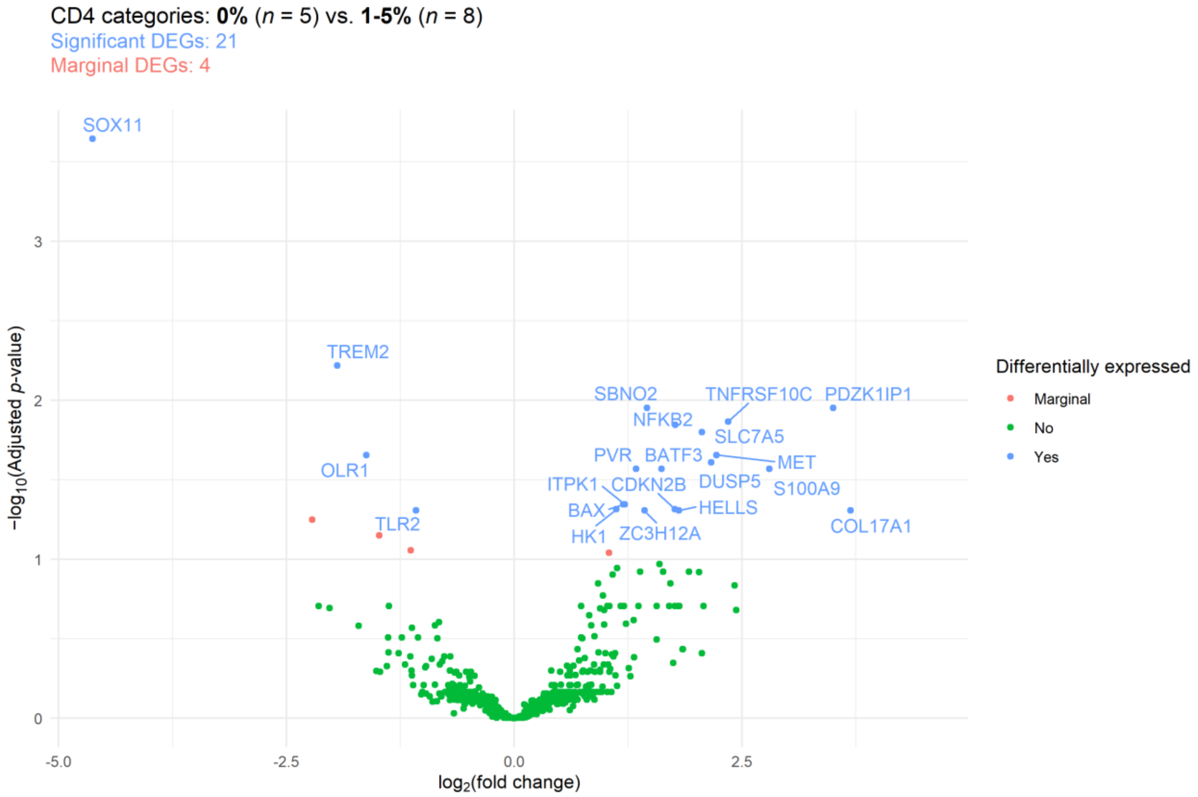

Figure S1. Differentially expressed genes (DEGs) between those ovarian cancer patients, whose tumor was negative (0%) and weakly (1-5%) stained for CD4+ T-lymphocyte infiltration. The false discovery rate method was used for *p*-value adjustment. *Reference category: patients with negative (0%) CD4+ T-lymphocyte infiltration.*

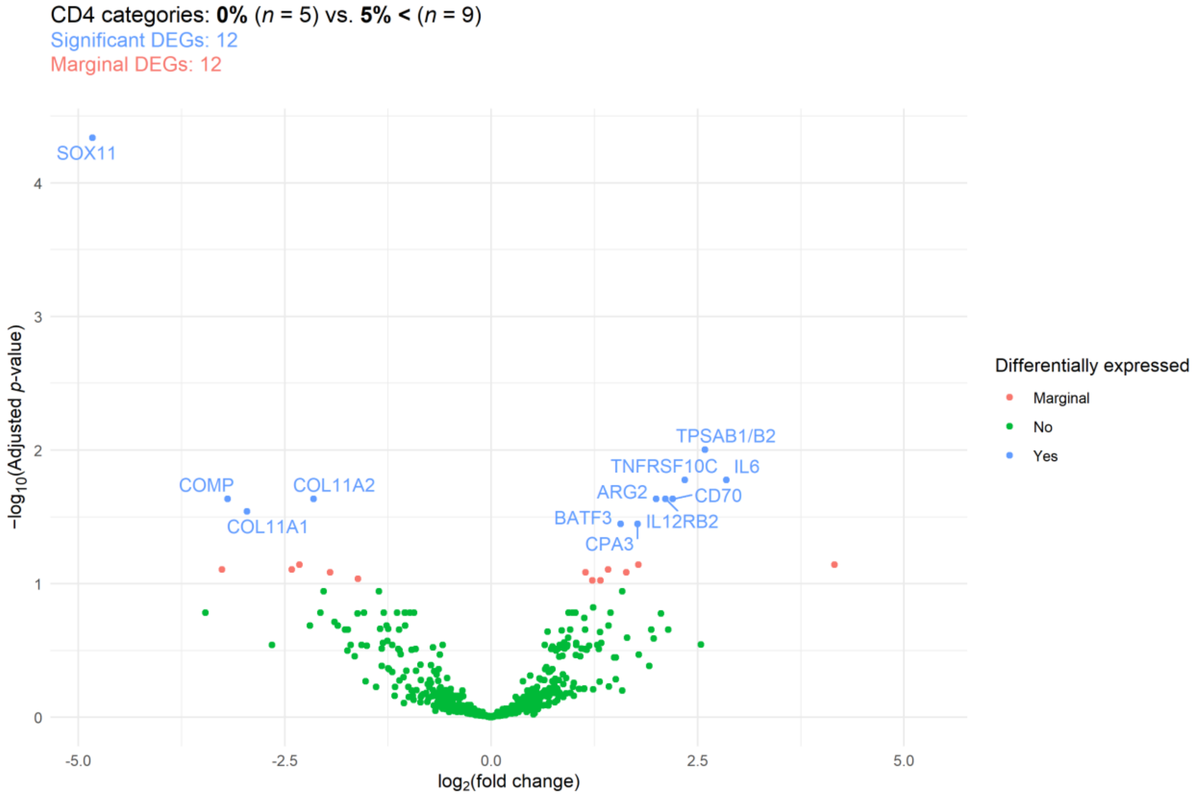

Figure S2. Differentially expressed genes (DEGs) between those ovarian cancer patients, whose tumor was negative (0%) and moderately/strongly (5% <) stained for CD4+ T-lymphocyte infiltration. The false discovery rate method was used for *p*-value adjustment. *Reference category: patients with negative (0%) CD4+ T-lymphocyte infiltration.*

† These authors contributed equally to this work.

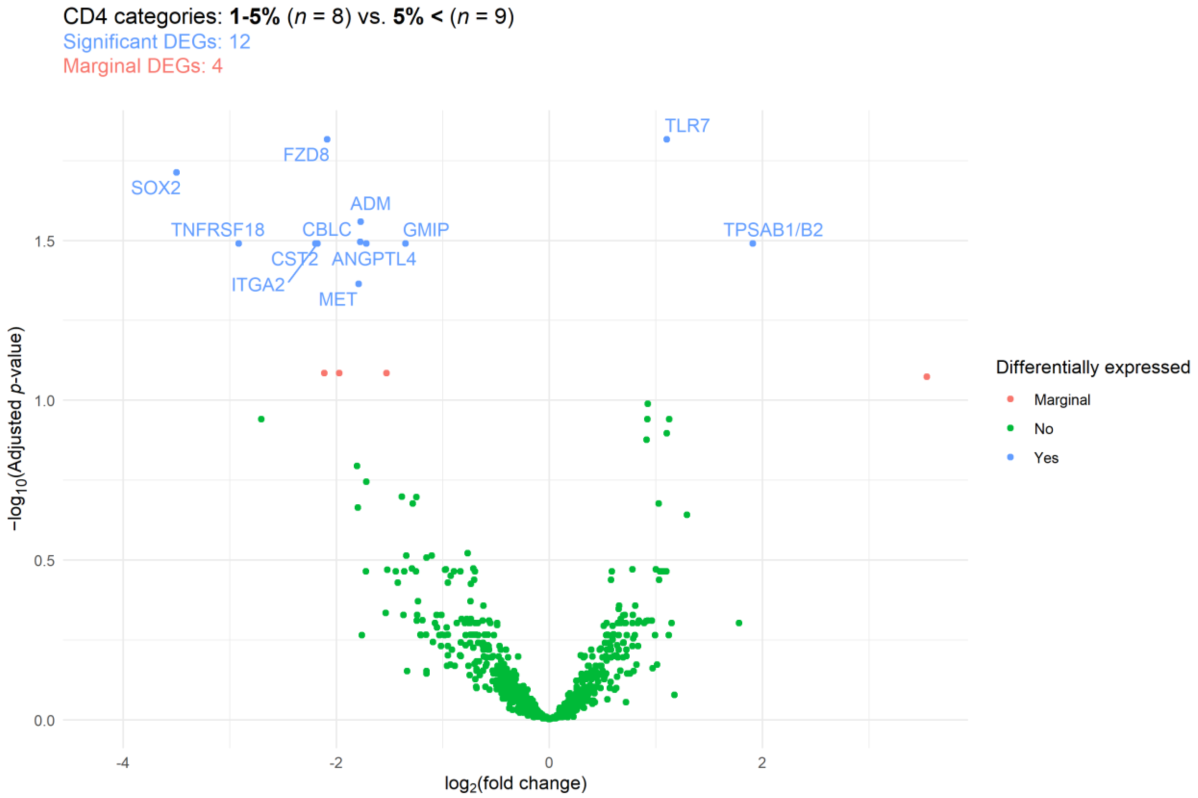

Figure S3. Differentially expressed genes (DEGs) between those ovarian cancer patients, whose tumor was weakly (1-5%) and moderately/strongly (5% <) stained for CD4^{+} T-lymphocyte infiltration. The false discovery rate method was used for p-value adjustment. *Reference category: patients with weak (1-5%) CD4^{+} T-lymphocyte infiltration.*

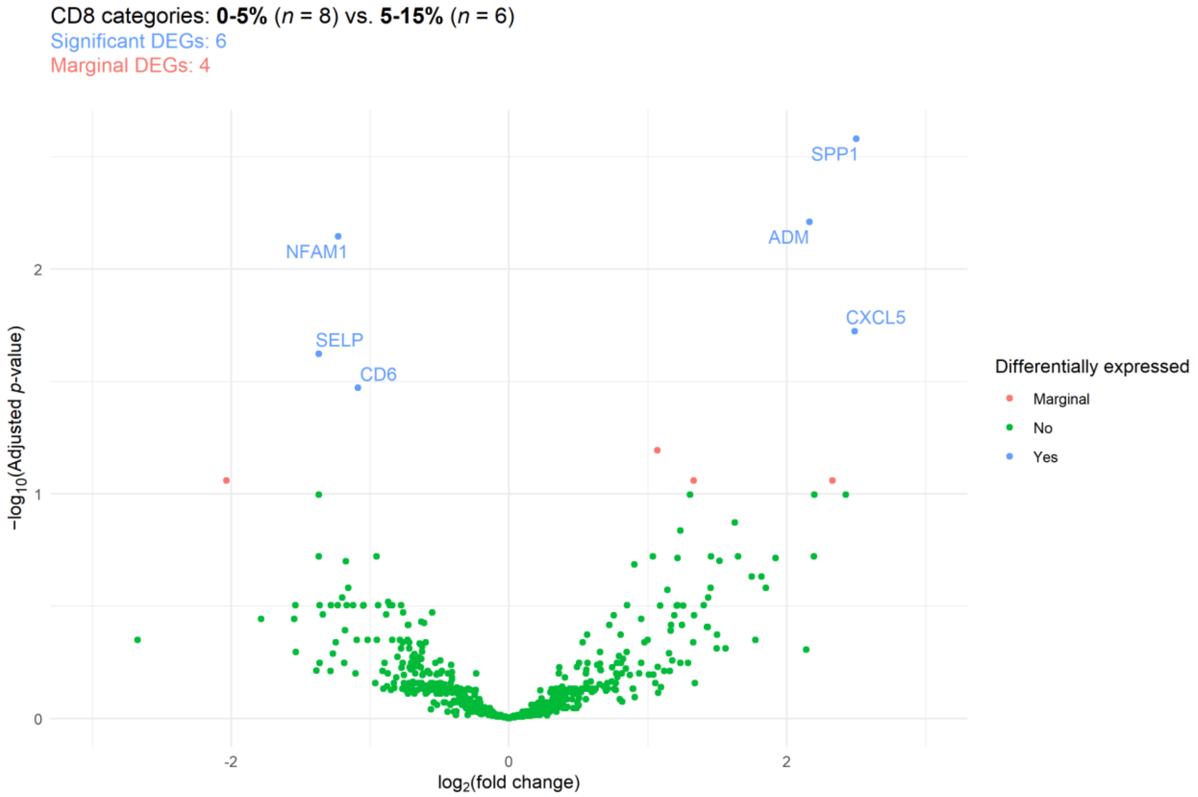

Figure S4. Differentially expressed genes (DEGs) between those ovarian cancer patients, whose tumor was negatively/weakly (0-5%) and moderately (5-15%) stained for CD8^{+} T-lymphocyte infiltration. The false discovery rate method was used for p-value adjustment. *Reference category: patients with negative/weak (0-5%) CD8^{+} T-lymphocyte infiltration.*

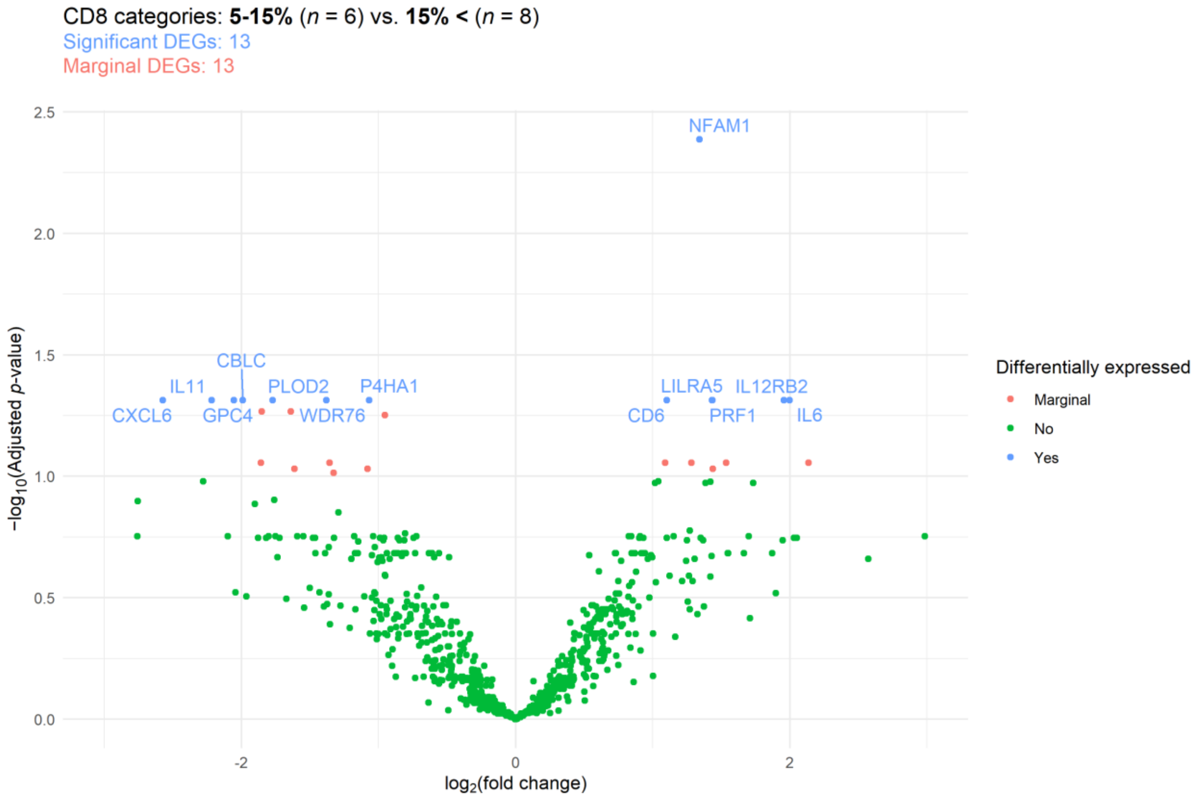

Figure S5. Differentially expressed genes (DEGs) between those ovarian cancer patients, whose tumor was moderately (5-15%) and strongly (15% <) stained for CD8+ T-lymphocyte infiltration. The false discovery rate method was used for *p*-value adjustment. *Reference category: patients with moderate (5-15%) CD8+ T-lymphocyte infiltration.*

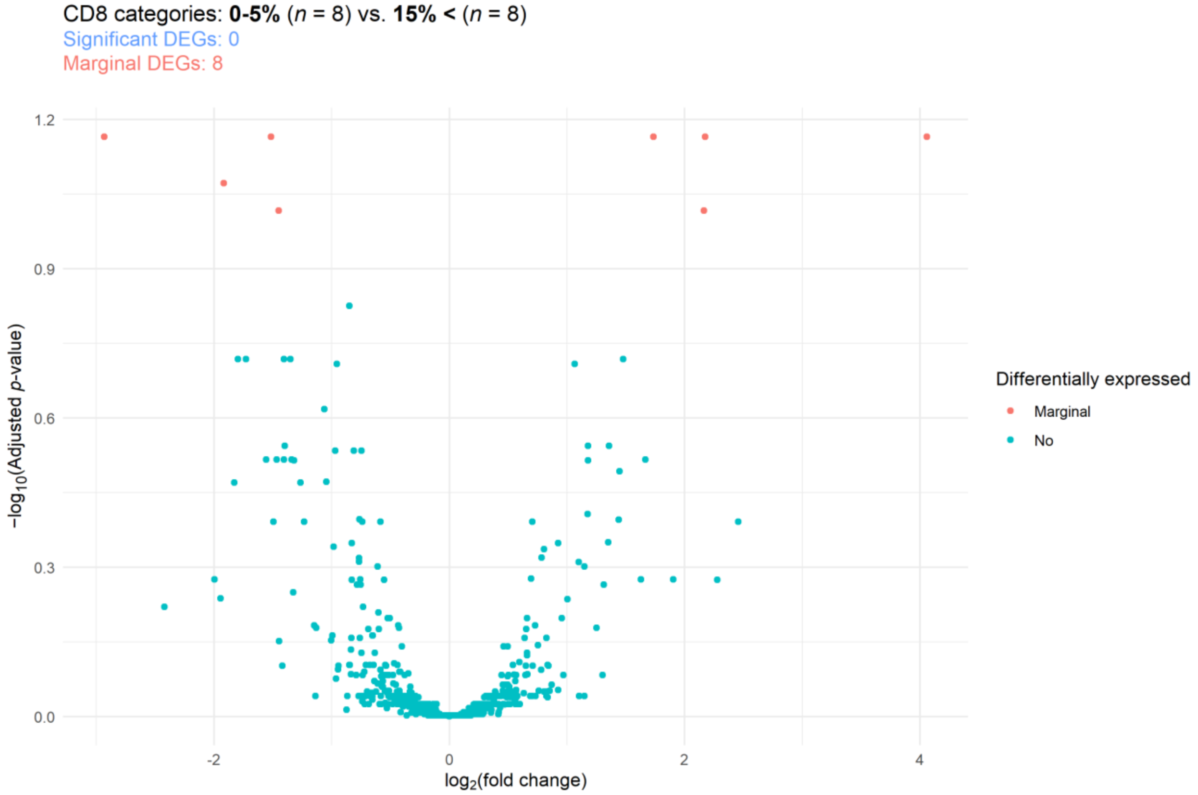

Figure S6. Differentially expressed genes (DEGs) between those ovarian cancer patients, whose tumor was negatively/weakly (0-5%) and strongly (15% <) stained for CD8+ T-lymphocyte infiltration. The false discovery rate method was used for *p*-value adjustment. *Reference category: patients with negative/weak (0-5%) CD8+ T-lymphocyte infiltration.*

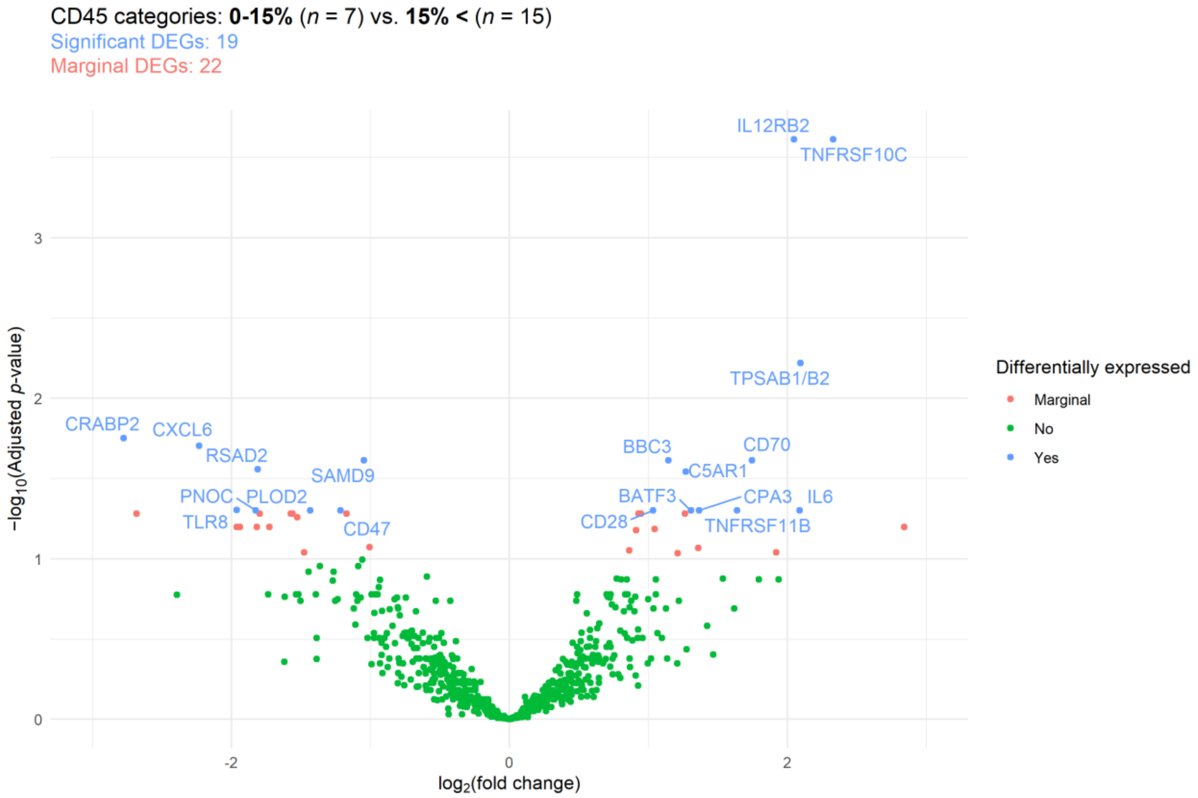

Figure S7. Differentially expressed genes (DEGs) between those ovarian cancer patients, whose tumor was negatively/weakly/moderately (0-15%) and strongly (15% <) stained for CD45+ T-lymphocyte infiltration. The false discovery rate method was used for *p*-value adjustment. *Reference category: patients with negative/weak/moderate (0-15%) CD45+ T-lymphocyte infiltration.*

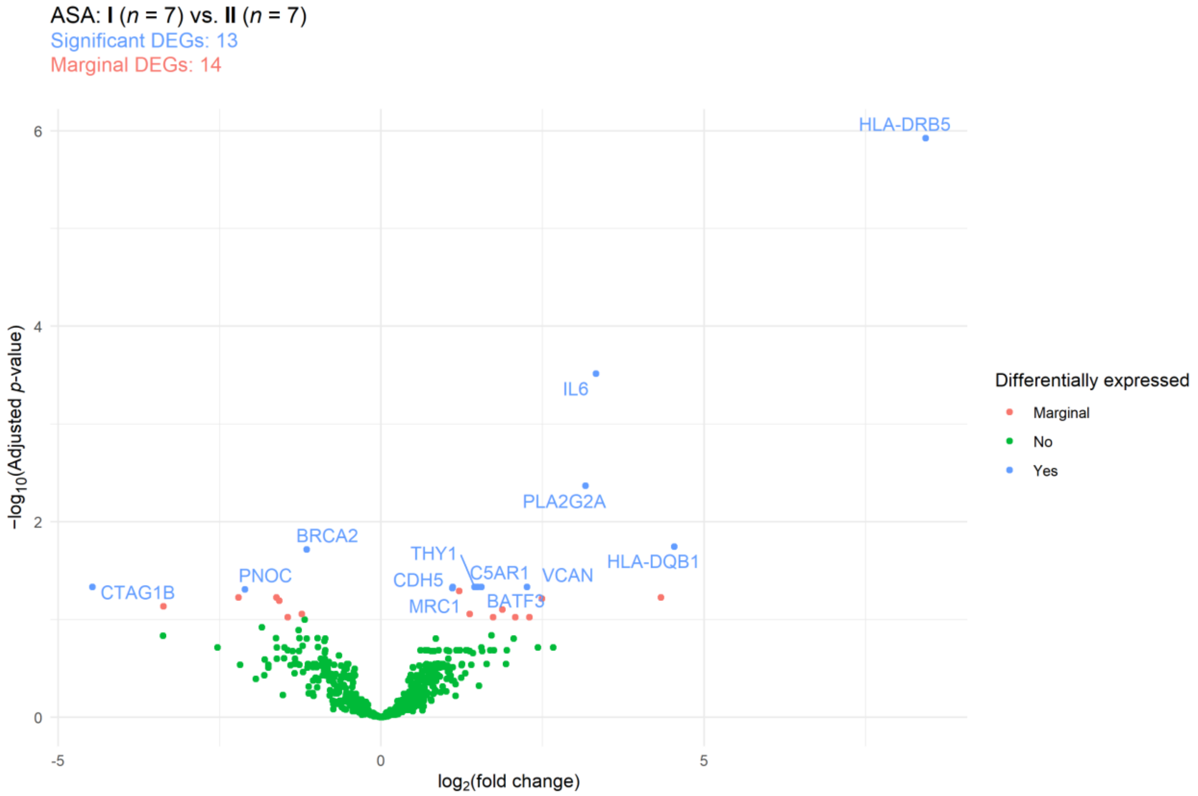

Figure S8. Differentially expressed genes (DEGs) between those ovarian cancer patients having an American Society of Anesthesiologists (ASA) performance score I vs. II. The false discovery rate method was used for *p*-value adjustment. *Reference category: patients with a T1 stage.*

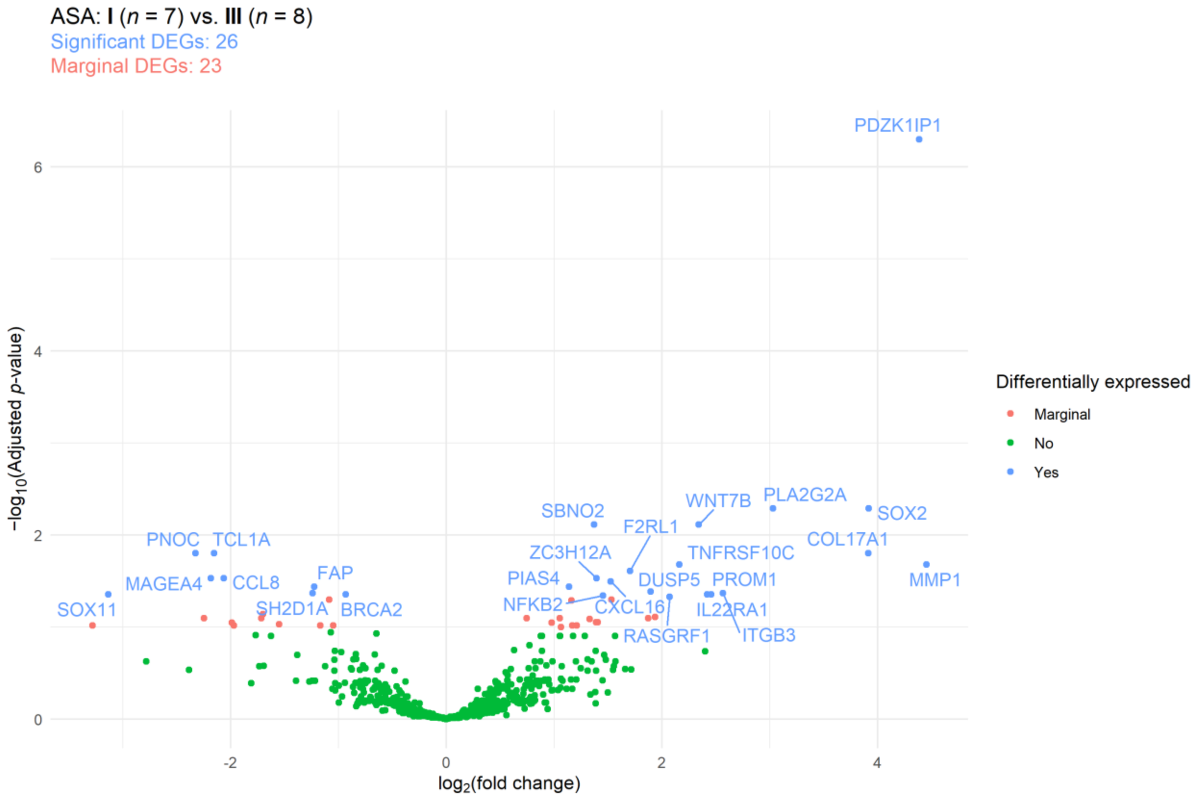

Figure S9. Differentially expressed genes (DEGs) between those ovarian cancer patients having an American Society of Anesthesiologists (ASA) performance score I vs. III. The false discovery rate method was used for *p*-value adjustment. *Reference category: patients with an ASA score of I.*

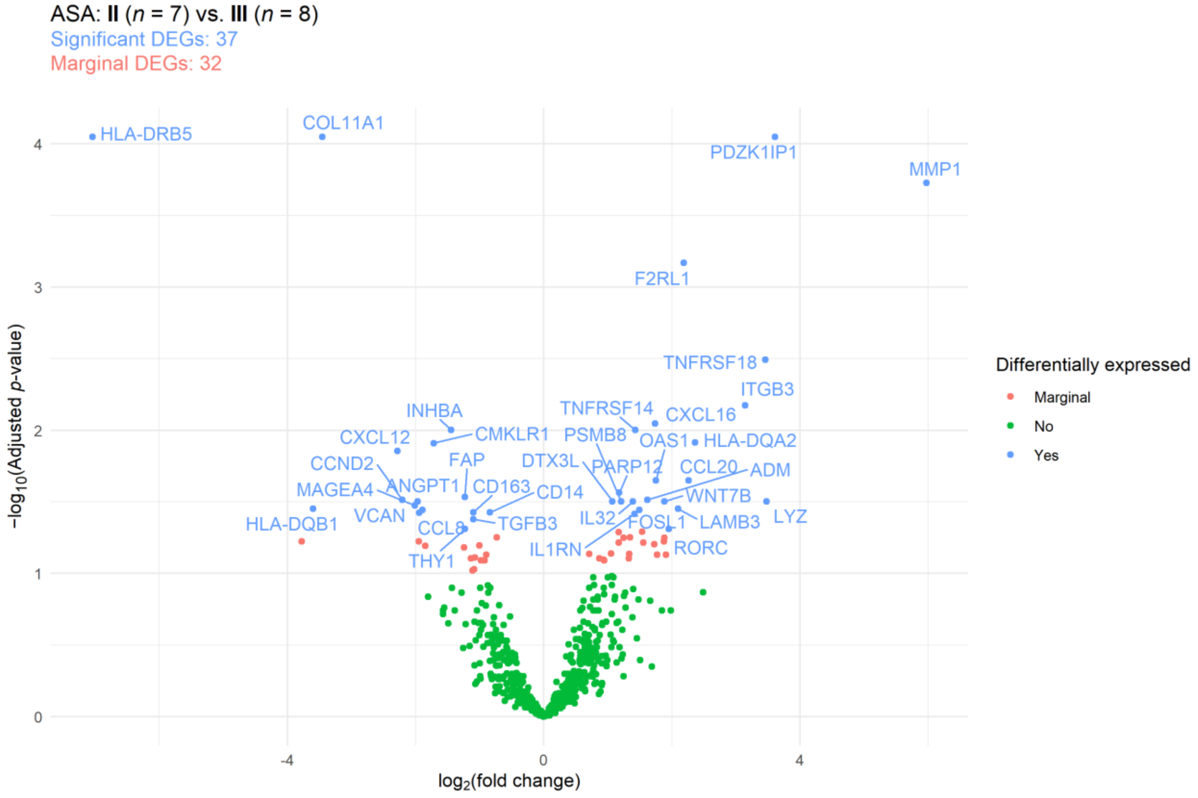

Figure S10. Differentially expressed genes (DEGs) between those ovarian cancer patients having an American Society of Anesthesiologists (ASA) performance score II vs. III. The false discovery rate method was used for *p*-value adjustment. *Reference category: patients with an ASA score of II.*

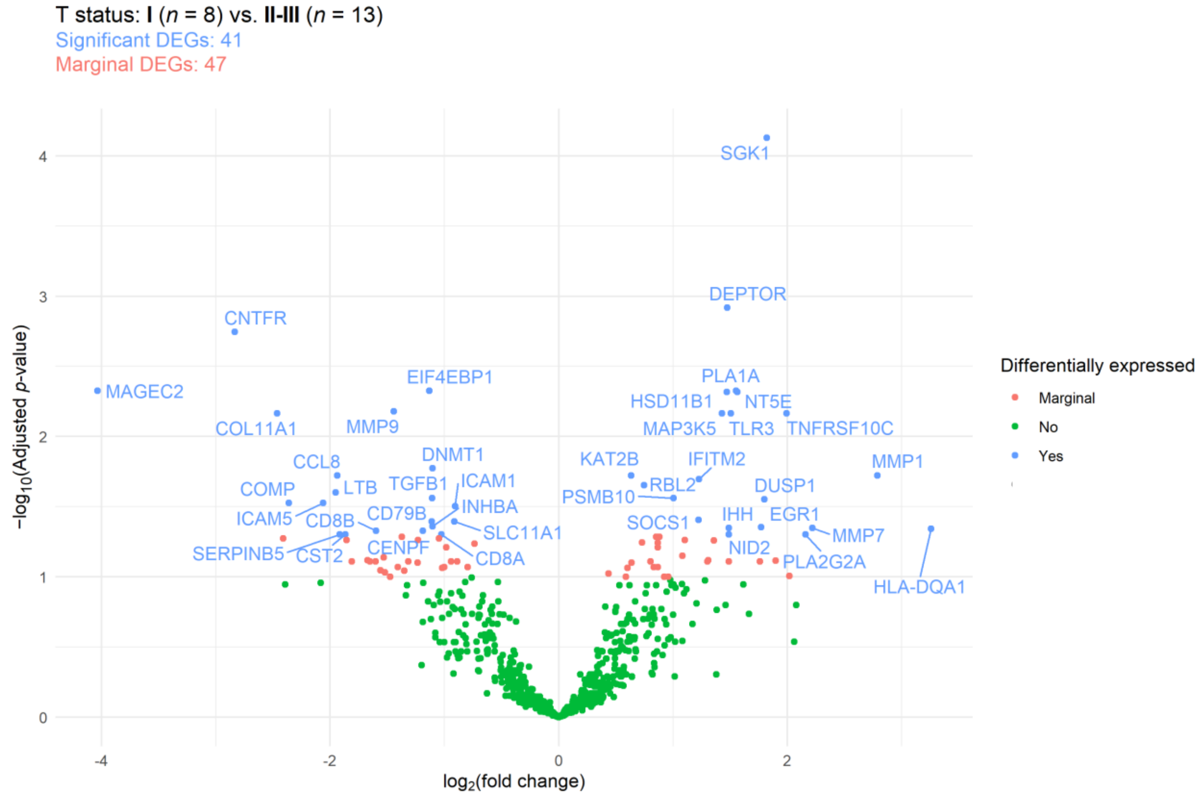

Figure S11. Differentially expressed genes (DEGs) between those ovarian cancer patients, whose tumor's T status was I vs. II-III. The false discovery rate method was used for p-value adjustment. *Reference category: patients with an ASA score of I.*

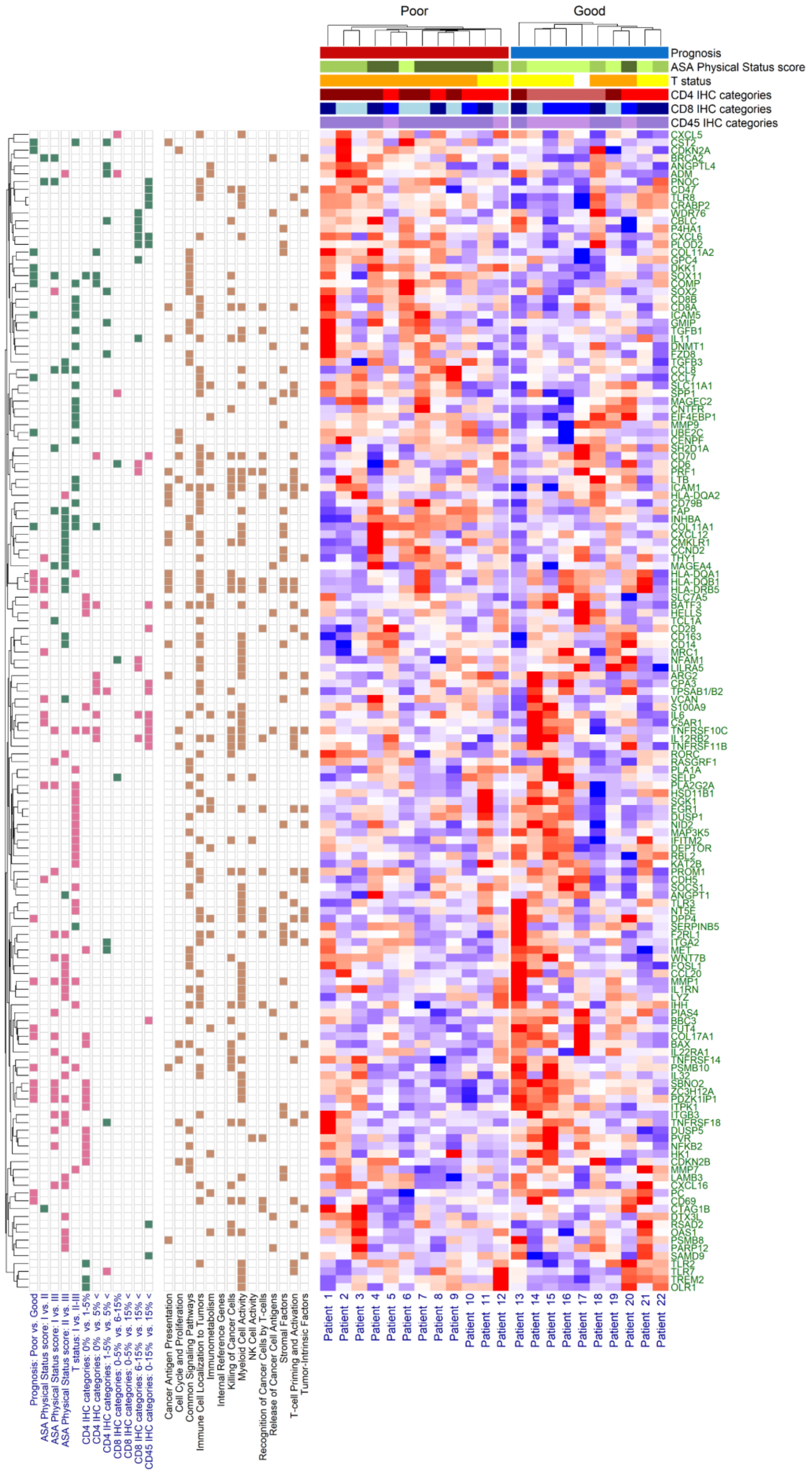

Figure S12. Heatmap of significantly different gene expressions. The green and pink boxes represent the down-expression and the up-expression of genes, respectively. Brown box shows functional enrichment annotation information of the differentially expressed genes. genes.

American Society of Anesthesiologists (ASA) performance scores: I (light green), II (green) and III (dark green); T status: I (yellow) and II-III (orange); CD4 IHC categories: 0% (light red), 1-5% (red) and 5% < (darkred); CD8 IHC

categories: 0-5% (light blue), 6-15% (blue) and 15% < (dark blue); CD45 IHC categories: 0-15% (light purple) and 15% < (purple).

Table S1. Result of the multivariate Cox regression investigating effect of the raw $CD4^+$, $CD8^+$, and $CD45^+$ tumor-infiltrating immune cell percentages and other clinical parameters over the overall survival of ovarian cancer patients.

Parameter	HR	95% CI	*p*-value
$CD4^+$ T-lymphocytes (%)	0.9381	0.8856 – 0.9933	0.0286
$CD8^+$ T-lymphocytes (%)	1.0238	0.9548 – 1.0977	0.5094
$CD45^+$ leukocytes (%)	1.0105	0.9441 – 1.0816	0.7631
Age (years)	1.0177	0.9696 – 1.0682	0.4776
Hemoglobin (g/L)	1.0239	0.9968 – 1.0518	0.0848
Platelet count (10^9/L)	1.0052	1.0014 – 1.0091	0.0077
Length of hospitalization (days)	1.1763	1.0491 – 1.3191	0.0054
Histology			
- Clear cell vs. Endometrioid	0.8876	0.0718 – 10.9743	0.9260
- Clear cell vs. Serous	0.3009	0.0311 – 2.9113	0.2997
- Clear cell vs. Mucinous	0.3113	0.0233 – 4.1554	0.3775
- Clear cell vs. Other types	0.6671	0.0585 – 7.6062	0.7444
- Endometrioid vs. Serous	0.3390	0.0932 – 1.2332	0.1006
- Endometrioid vs. Mucinous	0.3508	0.0712 – 1.7289	0.1980
- Endometrioid vs. Other types	0.7515	0.1765 – 3.1995	0.6992
- Serous vs. Mucinous	1.0348	0.2496 – 4.2896	0.9624
- Serous vs. Other types	2.2172	0.6464 – 7.6056	0.2055
- Mucinous vs. Other types	2.1426	0.4384 – 10.4710	0.3465
ASA performance score			
- I. vs. II.	27.8760	2.4091 – 322.5548	0.0077
- I. vs. III.	28.6674	2.0817 – 394.7840	0.0122
- I. vs. IV.	9.4239	0.5206 – 170.5966	0.1290
- II. vs. III.	1.0284	0.3730 – 2.8352	0.9567
- II. vs. IV.	0.3381	0.0585 – 1.9547	0.2258
- III. vs. IV.	0.3287	0.0608 – 1.7784	0.1965

ASA: American Society of Anesthesiologists; CI: confidence interval; HR: hazard rate.

Table S2. Clinical characteristics of those study participants, whose tumor specimens were selected for the NanoString gene expression analysis (n = 22). Two groups were created based on the survival time of the patients. 10 and 12 patients were enrolled into the good and poor prognosis groups, where the survival time was over and over 12 months, respectively.

Parameter	Poor prognosis group (n = 12)	Good prognosis group (n = 10)	*p*-value
Age (years)	57.58 ± 19.79	65.31 ± 13.75	0.4176
Weight (kg)	71.25 ± 10.06	73.90 ± 13.19	0.6915
No. of births	2.00 ± 1.28	1.40 ± 1.17	0.3263
No. of abortions	0.17 ± 0.39	0.20 ± 0.63	0.7823
$CD4^+$ T-lymphocytes (%)	2.75 ± 3.22	21.40 ± 17.75	0.0038
$CD8^+$ T-lymphocytes (%)	15.08 ± 13.63	13.30 ± 15.44	0.5518
$CD45^+$ leukocytes (%)	17.83 ± 16.09	28.60 ± 12.41	0.0465
Hemoglobin (g/L)	123.50 ± 11.94	124.10 ± 19.46	0.6434
Hematocrit (L/L)	0.38 ± 0.04	0.38 ± 0.07	0.7156
Platelet count (10^9/L)	389.92 ± 115.10	302.80 ± 167.44	0.0408
Carcinoembryonic antigen (ng/mL)	18.10 ± 30.70	259.54 ± 669.26	0.5656
Carbohydrate antigen 125 (U/mL)	1804.95 ± 2795.71	292.51 ± 311.22	0.2055
Duration of symptoms (months)	4.22 ± 4.29	4.45 ± 5.47	1.0000
Length of hospitalization (days)	7.91 ± 3.24	5.70 ± 1.57	0.0565
ASA performance score (I : II : III)	1 : 4 : 7<br>(8.3% : 33.3%: 58.3%)	6 : 3 : 1<br>(60.0% : 30.0% : 10.0%)	0.0217
Median survival (months)	4.37	not reached	–

ASA: American Society of Anesthesiologists.

Supplementary methods

Approach for tumor infiltrating lymphocyte assessment based on the Salgado 2015 criteria:

- ***TILs Assessment in Stromal Compartment:*** TILs were assessed in the stromal compartment to determine the percentage of stromal TILs. This evaluation involved calculating the area of stromal tissue, defined as the proportion of the intratumoral stromal area occupied by mononuclear inflammatory cells. Importantly, this assessment was not based on enumerating individual stromal cell counts but rather on quantifying the fraction of the total stromal area occupied by mononuclear inflammatory cells.
- ***Invasive Tumor Border Analysis:*** To ensure accuracy, TILs were exclusively evaluated within the confines of the invasive tumor. Areas outside the tumor border, as well as regions around Ductal Carcinoma In Situ (DCIS) and normal lobules, were systematically excluded from the analysis.
- ***Exclusion Criteria:*** Several exclusion criteria were applied during TILs assessment. These included disregarding TILs in tumor zones displaying crush artifacts, necrosis, regressive hyalinization, and those located within the previous core biopsy site.
- ***Inclusion of Mononuclear Cells:*** In line with established protocols, all mononuclear cells, encompassing lymphocytes and plasma cells, were included in the evaluation, while polymorphonuclear leukocytes were explicitly excluded from consideration.
- ***Tissue Section Preparation:*** For each patient, one tissue section measuring 4–5 μm in thickness and examined at magnifications of ×200–400 was utilized for TILs assessment. Full tissue sections were preferred whenever feasible. In cases of pretherapeutic neoadjuvant treatment, core biopsies were accepted for analysis, as validated post-neoadjuvant treatment TILs scoring methodologies were not available.
- ***Pathologist's Assessment:*** The assessment of TILs within the tumor area was conducted comprehensively by a trained pathologist. The focus was placed on evaluating the average TILs distribution within the tumor rather than concentrating solely on localized high-density areas, commonly referred to as "hotspots."
- ***Continuous Parameter Assessment:*** TILs were assessed as a continuous parameter. The percentage of stromal TILs served as a semiquantitative measure indicating the extent of mononuclear inflammatory cell infiltration within the stromal tissue. To ensure precision, the assessment accounted for the variable growth patterns of lymphocytes, acknowledging that lymphocytes do not typically form solid cellular aggregates. Thus, even in cases designated as "100% stromal TILs," some interstitial tissue space between individual lymphocytes was considered.
- ***Clinical Threshold Determination:*** At the present stage, no formal recommendations were made regarding clinically relevant TIL thresholds. The primary emphasis was placed on establishing a robust and validated assessment methodology. "Lymphocyte-predominant breast cancer" was employed as a descriptive term to characterize tumors with a higher lymphocyte presence than tumor cells, with thresholds for such cases varying between 50% and 60% stromal lymphocytes. Pathologists were encouraged to report TIL scores in as much detail as they deemed appropriate based on their assessment.
